# Isotopic systematics of zircon indicate an African affinity for the rocks of southernmost India

**DOI:** 10.1038/s41598-020-62075-y

**Published:** 2020-03-25

**Authors:** Chris Clark, Alan S. Collins, Richard J. M. Taylor, Martin Hand

**Affiliations:** 1 School of Earth & Planetary Sciences, Curtin University, GPO Box 1987, Perth, WA 6845 Australia; 2 Department of Earth Sciences, the University of Adelaide, Adelaide, SA 5005 Australia

**Keywords:** Geochemistry, Precambrian geology, Petrology

## Abstract

Southern India lies in an area of Gondwana where multiple blocks are juxtaposed along Moho-penetrating structures, the significance of which are not well understood. Adequate geochronological data that can be used to differentiate the various blocks are also lacking. We present a newly acquired SIMS U–Pb, Lu–Hf, O isotopic and trace element geochemical dataset from zircon and garnet from the protoliths of the Nagercoil Block at the very tip of southern India. The data indicate that the magmatic protoliths of the rocks in this block formed at c. 2040 Ma with Lu–Hf, O-isotope and trace element data consistent with formation in a magmatic arc environment. The zircon data from Nagercoil Block are isotopically and temporally distinct from those in all the other blocks in southern India, but remarkably correspond to rocks in East Africa that are exposed on the southern margin of the Tanzania–Bangweulu Block. The new data suggest that the tip of southern India has an African affinity and a major suture zone must lie along its northern margin. All of these blocks were finally brought together during the Ediacaran-Cambrian amalgamation of Gondwana where they underwent high to ultrahigh temperature metamorphism.

## Introduction

Southern peninsular India is the meeting place of two major strands of the East African Orogen (EAO) and has been referred to as the “Gondwana Junction” e.g. Santosh, *et al*.^1^. However, the southernmost tip of India (the Nagercoil Block) remains an enigma, as it has been included as part of Proterozoic India^2,3^, with correlation and connection to Africa^4,5^, or Sri Lanka^6,7^.

Understanding the crustal framework and sequence of events that occurred during the final stages of Gondwana assembly in this region is important for a number of reasons. Firstly, considerable controversy surrounds the tectonic evolution of the India-Africa collision, the largest of the Gondwana-forming orogens (contrast the conclusions of Collins, *et al*.^8^ and Plavsa, *et al*.^9^ with Tucker, *et al*.^2^ and Boger *et al*.^3^). Secondly, this area forms one of the largest exposed regions of extreme crustal metamorphism found on the planet^8^, the drivers for which have been challenging to model^10,11^. Initial parameters in developing models to explain this process requires knowledge of the lithospheric framework, something that in the EAO is still unresolved. Here we present new SIMS U–Pb zircon, Lu–Hf and O isotopic data that strongly point to the pre-Gondwana provenance of the Nagercoil Block and constrain the lithospheric framework of this significant part of the Gondwana amalgam.

## Geological setting of the Nagercoil Block

**Figure 1:**
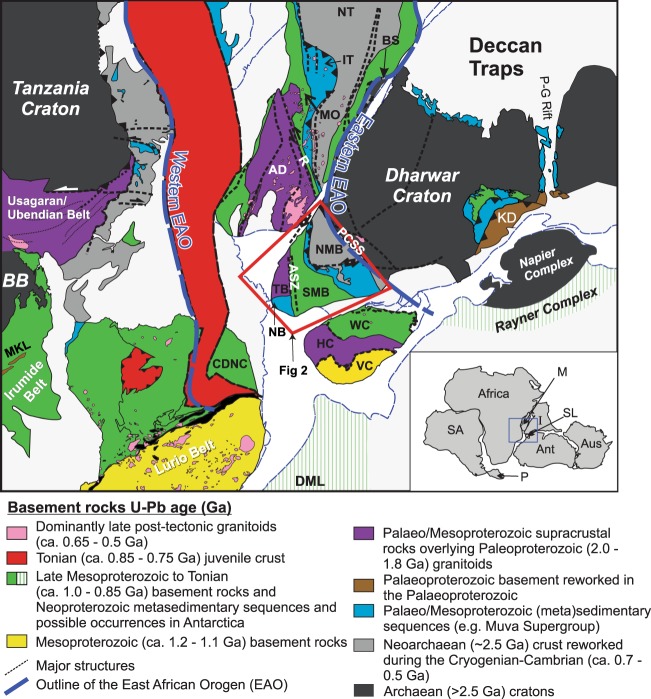
Geological map of Gondwanan terranes. Gondwana reconstruction after Collins and Pisarevsky (2005). The major geological blocks, formations, and structures include: NT—Antananarivo block; IT—Itermo Group; MO—Molo Group; AD—Androyen block; VO—Vohibory block; CDNC—Cabo Delgado nappe complex; BS—Betsimisaraka suture; R—Ranotsara shear zone; PCSS—Palghat-Cauvery shear system; ASZ—Achankovil shear zone; the eastern and western limits of East African orogen (EAO); NMB—northern Madurai block; SMB— southern Madurai block; TB + NB—Trivandrum and Nagercoil blocks; WC—Wanni complex; HC—Highland complex; DML—Dronning Maud Land; KD—Krishnagiri domain; VC—Vijayan complex; MKL - Mkushi-Luwalizi gneisses; BB - Bangeweulu Bloc. The inset shows a tight fit of Gondwana fragments: M—Madagascar, SL—Sri Lanka; I— India; P—Patagonia; SA—South America; Ant—Antarctica; Aus—Australia. Please see text for references.

The assembly of the supercontinent Gondwana was the result of a series of collisions between different cratonic blocks resulting on one of the most significant orogenic belts in Earth history, the Ediacaran–Cambrian East African Orogen^12^. In plate reconstructions of this period India was the final cratonic block to accrete to the margin of Gondawana and this occurred between 0.61–0.53 Ga^12–14^. The final assembly of Gondwana resulted in the juxtaposition of southern India and with regions of Madagascar, Sri Lanka, eastern Africa and Antarctica (Fig. 1). The southern tip of India (the Nagercoil block) is a crucial location in this reconstruction as it is where a number of the orogenic belts that resulted from the amalgamation of the cratonic blocks join (Fig. 1).

**Figure 2:**
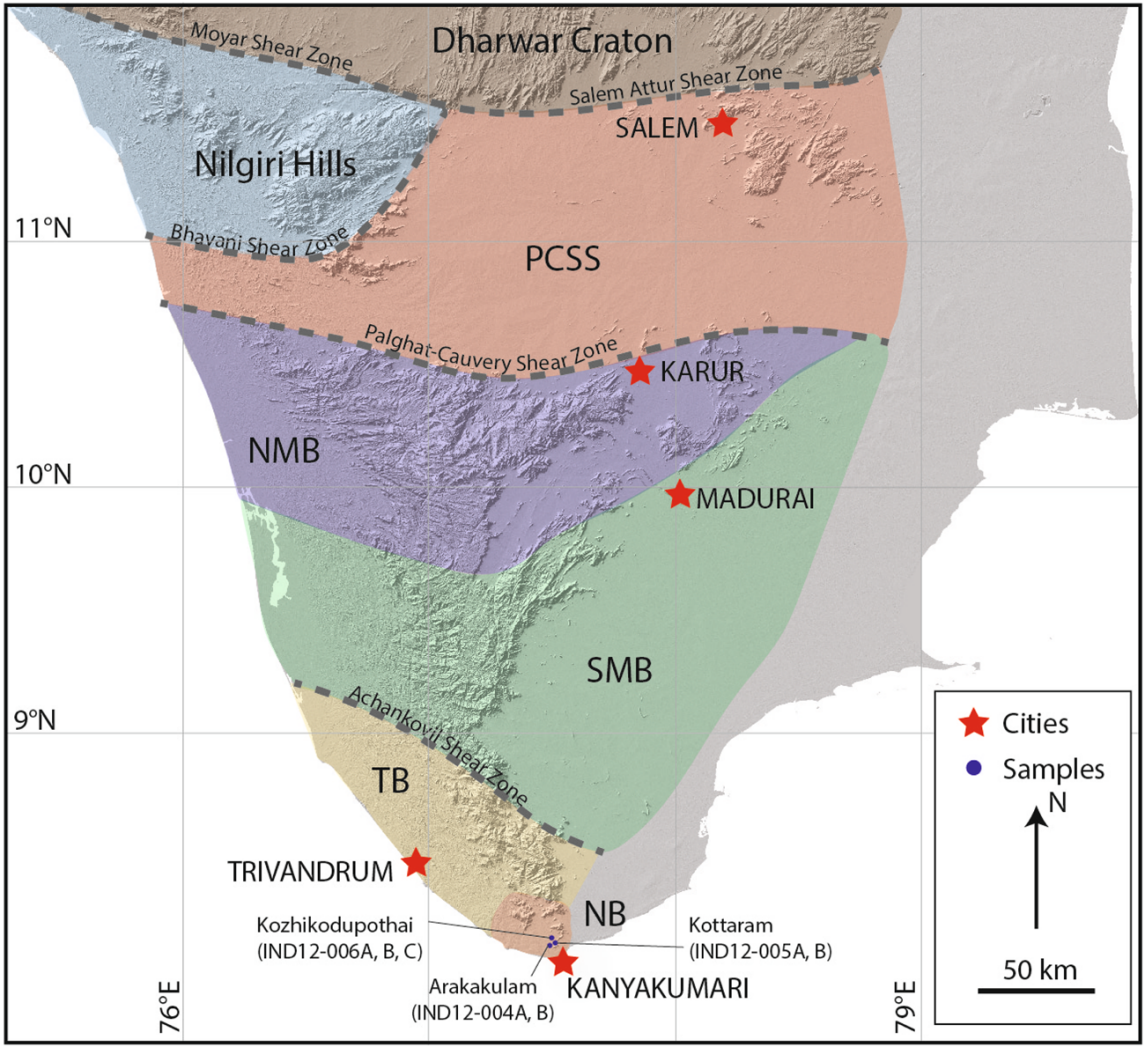
Map of the Southern Granulite Terrane showing the different tectonic units and the major structures/shear zones separating blocks. Nagercoil Block and sample locations shown in the southernmost part of peninsular India.

The southern tip of India is dominated by massive charnockites (here we use the term charnockite to indicate an orthopyroxene-bearing felsic gneiss, no genetic link to the origin of the orthopyroxene, whether igneous or metamorphic, is inferred) that previous studies have identified to be distinct from the adjoining Trivandrum and Madurai Blocks and is referred to as the Nagercoil Block^15,16^ (Fig. 2). Geochronological and whole rock isotopic investigations of the Nagercoil Block charnockites reveal similarities in the age and geochemical character to some of the protoliths of the Trivandrum Block^7,17–20^. By contrast, Santosh *et al*.^1^ presented a scenario where the massive charnockites of the Nagercoil Block were generated in a Neoproterozoic arc system. Santosh *et al*. suggest that the magmas were generated immediately prior to the amalgamation of Gondwana along a Pacific-type subduction margin. This argument was supported by the adakitic geochemical signatures of the Nagercoil charnockites and which are similar to the Neoproterozoic arc-related igneous rocks that have been identified to the north in the southern Madurai Block^21^ (Fig. 2). In a geochemically focused study by Rajesh *et al*.^22^ it is suggested that the granitic plutons that form the protoliths to the exposed charnockites were generated by melting of hydrous basalts in a subduction setting. Although Rajesh *et al*.^22^ were equivocal about the timing of this melting and what the proposed arc was related to. The formation of the characteristic charnockite assemblages present in the exposed Nagercoil Block gneisses is a result of the high-grade metamorphism during the Neoproterozoic–Cambrian orogenic event, this idea will be further tested in this study through the collection of rare earth elements in zircon rims and garnet^23,24^. Johnson *et al*.^25^ have constrained the pressure–tempetrature–time *(P–T–t)* history of the Nagercoil Block rocks to be comparable to those recorded within the adjoining Madurai and Trivandrum Blocks^10,26,27^.

## Results

Samples were collected from three locations within the Nagercoil Block to constrain the age of magmatism and metamorphism. The field relationships, petrography, results for SHRIMP U–Pb, Lu–Hf and oxygen isotope zircon analyses and LA-ICPMS rare earth element (REE) compositions of zircon, garnet and orthopyroxene are reported below. Complete data sets are reported in Supplementary Tables S1–S5.

### Petrographic descriptions of samples and field relationships

#### Arakakulam

**Figure 3:**
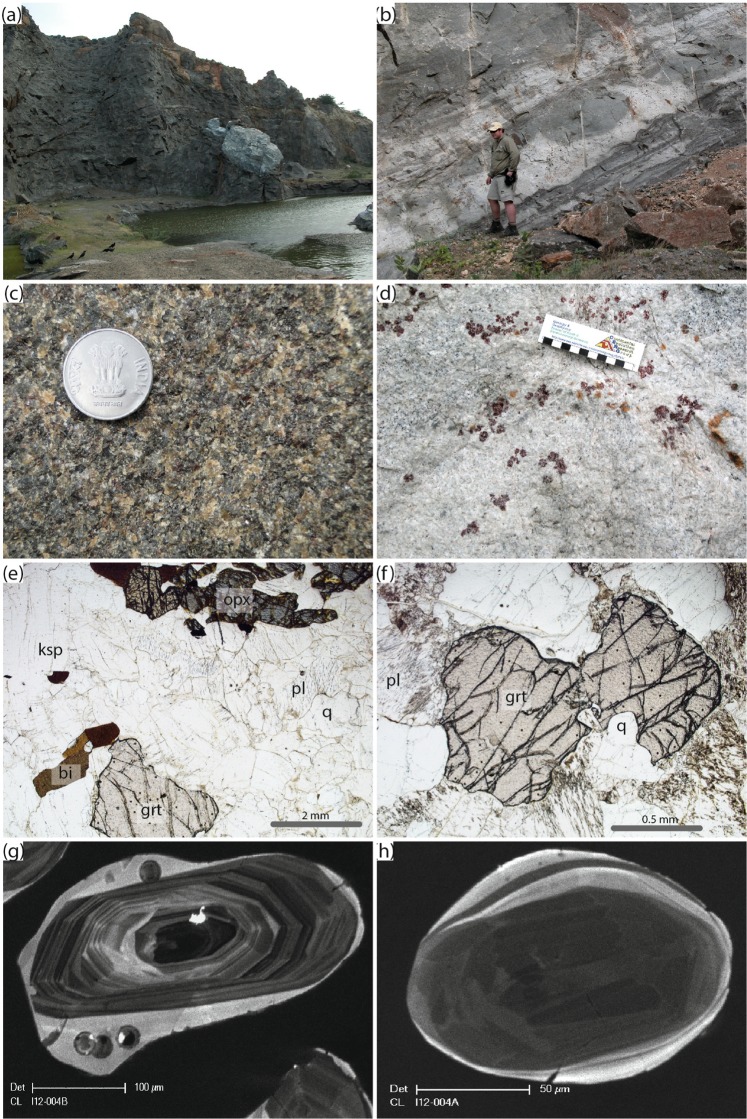
Field and petrographic relationships at Arakkakulam quarry. (**a**) Field photo of the Arakakulam quarry with an exposed calcsilicate lens. (**b**) Discordant garnet-bearing leucogranites within the charnockite. (**c**) Field photo of the equigranular garnet-bearing charnockite. (**d**) Field photo of the garnet-bearing leucogranite with large (up to 2 cm) garnets. (**e**) Photomicrograph of the garnet-bearing charnockite (sample Ind12-04b) with garnet (grt), orthopyroxene (opx), biotite (bi) in a framework of plagioclase (pl) and quartz (q). Note that the orthopyroxene is being partially altered to amphibole. (**f**) Photomicrograph of garnet-bearing leucogranite (sample Ind12-05A) showing a euhedral garnet within a framework of plagioclase and quartz. (**g**) Cathodoluminesence (CL) image of at typical oscillatory-zoned (igneous) zircon core mantled by CL-bright (metamorphic) rim from the garnet-bearing charnockite. (**h**) CL image of a sector zoned zircon from the garnet-bearing leucogranite.

The quarry at Arakakulam contains exposures dominated by garnet-absent charnockite and with subordinate garnet-bearing charnockite (Fig. 2). The charnockites are weakly foliated with the foliation defined by orthopyroxene, garnet and when present, biotite. Garnet occurs in the charnockite adjacent to calc-silicate enclave at Arakkakulam quarry. A garnet-leucogranite intrudes the charnockite at the southern end of the quarry. A summary of the field and petrographic relationships are presented in Fig. 3a–g.

#### Kottaram

**Figure 4:**
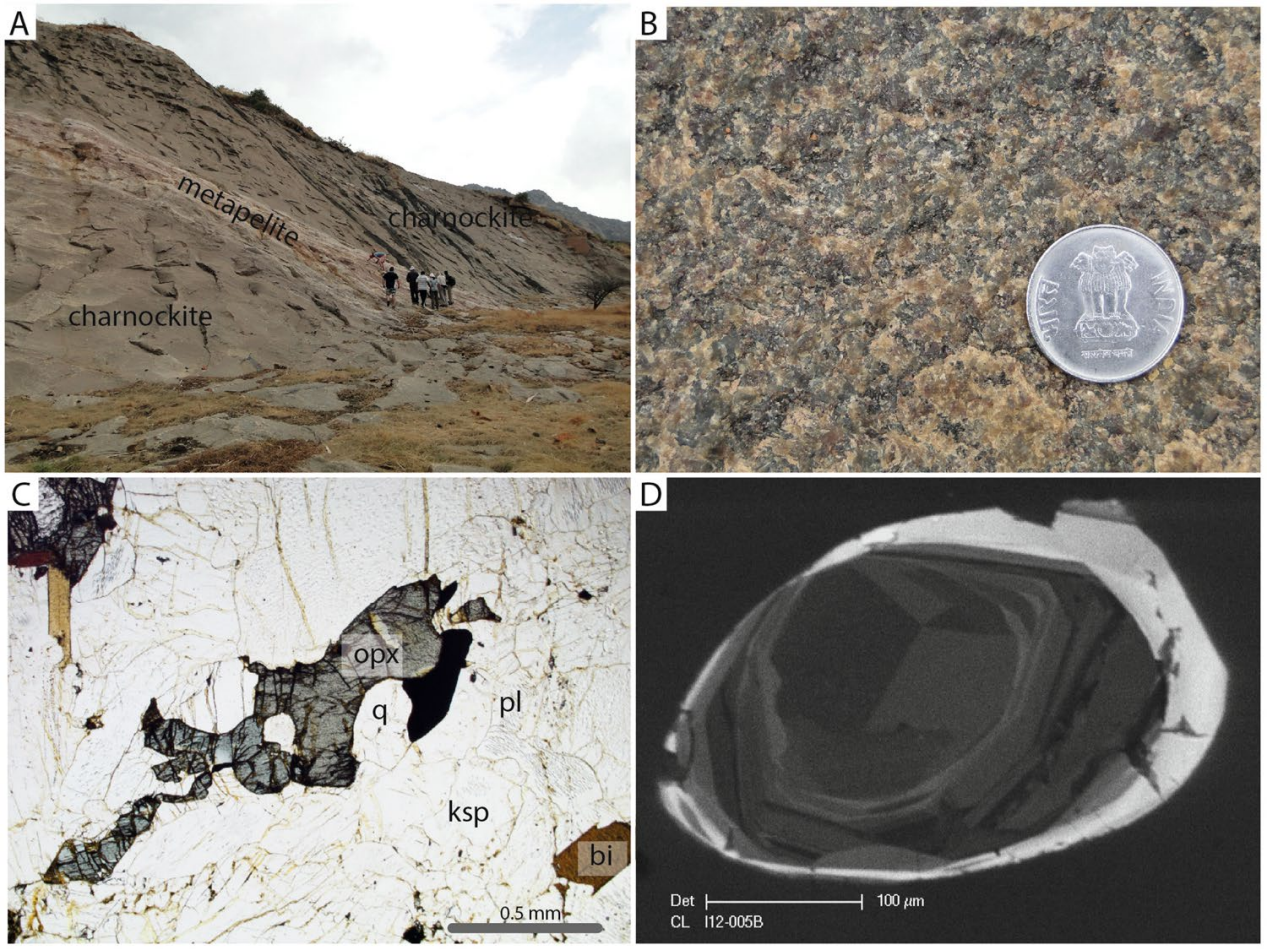
Field and petrographic relationships at Kottaram quarry. (**A**) Photo of the quarry at Kottaram illustrating the metapelitic raft enclosed within the massive charnockite. (**B**) Field photo of the equigranular massive charnockite. (**C**) Photomicrograph of the charnockite (sample Ind12-05A) with orthopyroxene (opx), biotite (bi) in a framework of plagioclase (pl), K-feldspar (ksp) and quartz (q). Note that the orthopyroxene is unretrogressed in comparison to the previous location. **(D**)) Cathodoluminesence (CL) image of at typical oscillatory-zoned (igneous) zircon core mantled by CL-bright (metamorphic) rim from the charnockite.

Charnockite bands are associated with ~20 m long metapelitic bands, charnockite proximal to the metapeltic bands and extending up to several metres from the contact contain higher modal proportions of garnet. The boundary between the metapelite raft and the charnockite is quite diffuse suggesting partial assimilation of metapelite during either the incorporation of the metapelite into the original granitic magma as a raft or during the high-temperature metamorphism and associated partial melting during the Neoproterozoic-Ediacaran. The garnet-enriched charnockite zone has a reddish color and extends up to ~10 m from the metapelite. At a distances greater than 10 m from the contact the rock is a homogeneous green-grey equigranular garnet-absent charnockite that shows no evidence of a pervasive foliation. At the eastern end of the quarry a sub-horizontal undeformed mafic dyke intrudes the charnockite. A summary of the field and petrographic relationships are shown in Fig. 4a–d.

#### Kozikhodupothai

**Figure 5:**
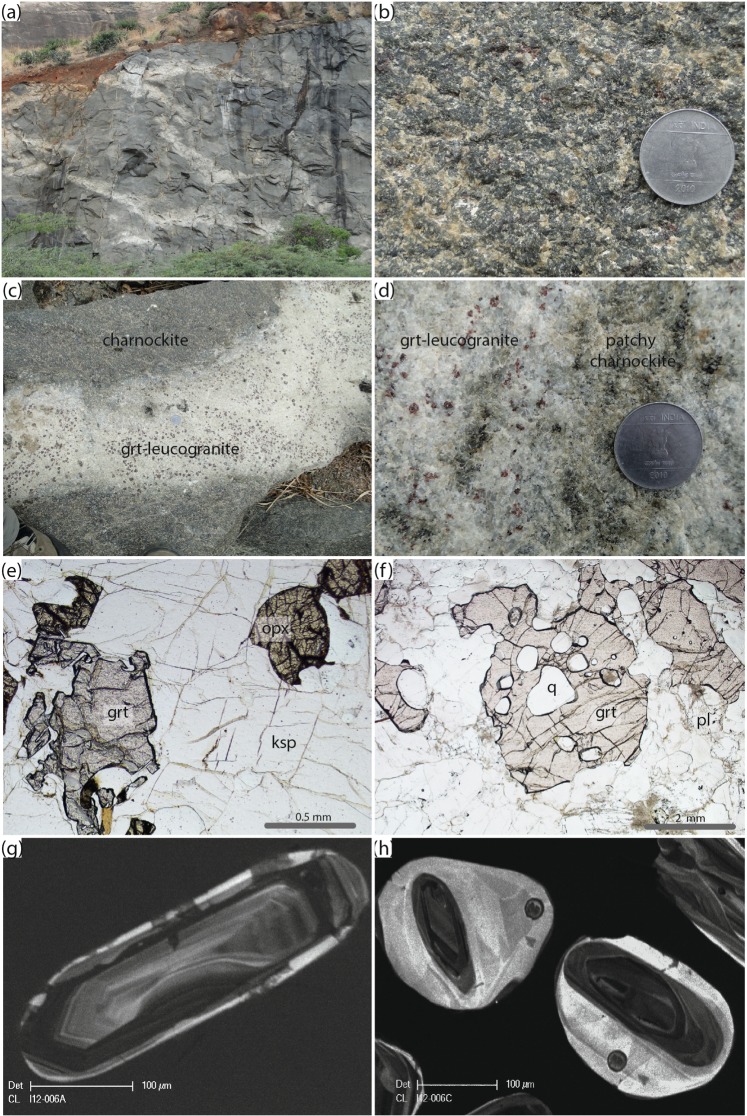
Field and petrographic relationships at Kozikhodupothai quarry. (**a)** Field photo of the Kozikhodupothai quarry showing the massive charnockite cross-cut by a series of garnet bearing leucogranites (**b**) Field photo of the equigranular garnet-bearing charnockite. (**c**) Discordant garnet-bearing leucogranites within the charnockite. (**d**) Field photo of the garnet-bearing leucogranite being overprinted by a discrete charnockite patch. (**e**) Photomicrograph of the garnet-bearing charnockite (sample Ind12-06a) with garnet (grt) and orthopyroxene (opx) in a framework of K-feldspar (ksp) and quartz (q). (**f**) Photomicrograph of garnet-bearing leucogranite (sample Ind12-05b) showing a euhedral garnet with large quartz inclusions typical of magmatic garnets within a framework of plagioclase and quartz. (**g**) Cathodoluminesence (CL) image of at typical oscillatory-zoned (igneous) from the garnet-bearing charnockite (Ind-006a). (**h**) Image of a typical oscillatory-zoned (igneous) zircon cores mantled by CL-bright (metamorphic) rims from the garnet-bearing leucogranite with patchy charnockite (Ind-006c).

Two charnockite styles, a garnet-absent and garnet-bearing, occur at Kozokodupothai, with the garnet-absent variety being the dominant type. The charnockites are cross-cut by an extensive network of garnet-bearing leucogranites. One of these granites contains evidence for the development of incipient charnockite. A summary of the field and petrographic relationships are shown in Fig. 5a–g.

### SHRIMP U-Pb and trace element data

#### Arakakulam

**Figure 6:**
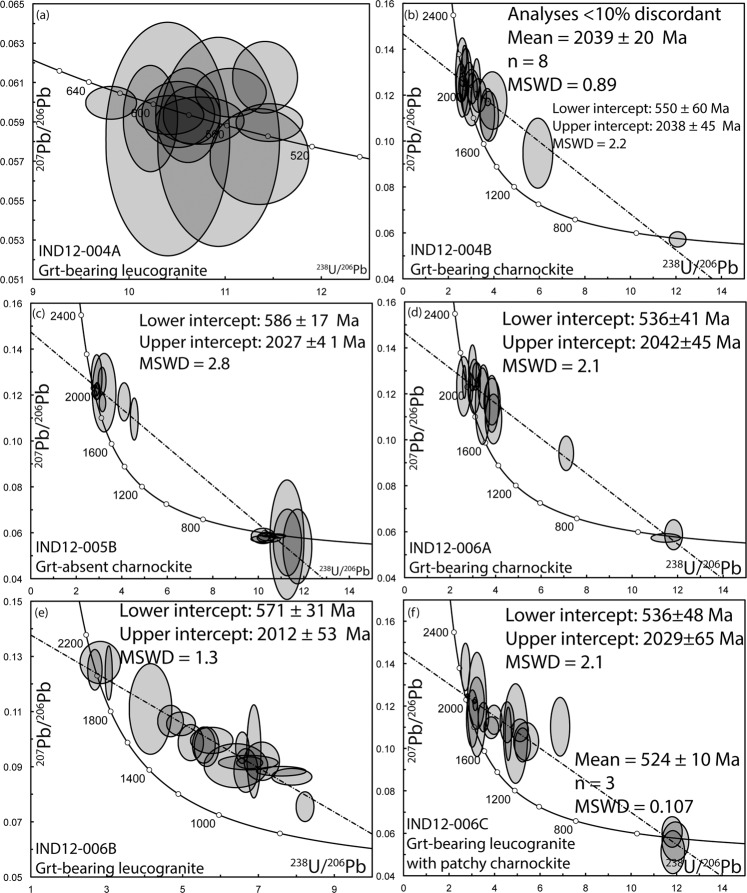
Tera-Wasserburg concordia diagram for zircon grains. **(a**) IND12-004A, (**b**) IND12-004B, (**c**) IND12-005B, (**d**) IND12-006A, (**e**) IND12-006B and (**f**) IND12-006C. Uncertainty ellipses are 2σ. Ages along the concordia curve are in Ma. Data tabulated in Supplementary Table S1.

**Figure 7:**
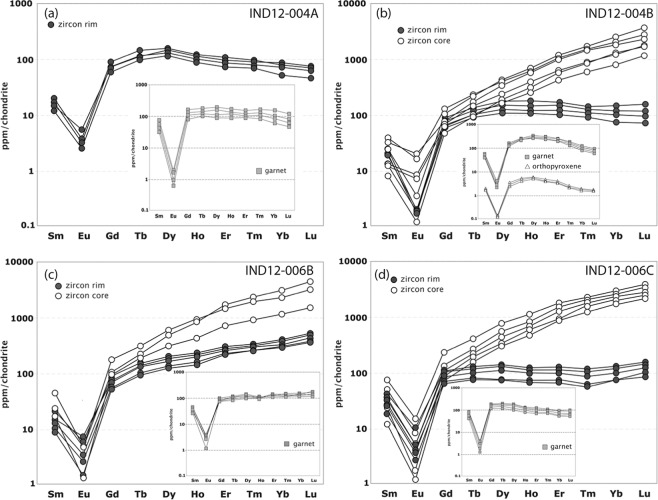
Chondrite normalised rare earth element (REE) plots. (**a**) IND12-004A, (**b**) IND12-004B, (**c)** IND12-006B, (**d**) IND12-006C. Data tabulated in Supplementary Tables S2 and S3.

Two samples (a garnet bearing charnockite and a cross-cutting garnet-leucogranite) were analysed at this location. Zircon grains from the garnet bearing charnockite (IND12–004B) yielded an upper intercept age of 2038 ± 45 Ma (MSWD = 2.2, n = 17; Fig. 6b) equivalent to the weighted ^207^Pb/^206^Pb average age of the least discordant analyses (<10% discordant) of 2039 ± 20 Ma (MSWD = 0.89, n = 8). A single analysis at the rim of a zircon from this sample gave a ^206^Pb/^238^U age of 515 ± 12 Ma that is within uncertainty of the lower intercept age of 550 ± 60 Ma (Fig. 6b). The REE analysis of zircon, garnet and orthopyroxene suggests that the 515 Ma zircon rims were in equilibrium with the garnet and orthopyroxene, whereas the oscillatory-zoned cores show typical igneous patterns and are not in equilibrium with the garnet (Fig. 7b). The discordant garnet leucogranite (IND12-004A) gave a single population of zircon with individual ^206^Pb/^238^U spot ages ranging between 590 Ma and 540 Ma (Fig. 6a). The REE analyses of garnet and zircon from this sample are suggestive of equilibrium between the two minerals (Fig. 7a).

#### Kottaram

A garnet-absent charnockite (IND12-005B) was sampled at this locality. Analyses of the oscillatory-zoned cores of zircon from the charnockite yield a discordia with an upper intercept of 2027 ± 41 Ma and a lower intercept of 585 ± 17 Ma (Fig. 6c) (MSWD = 2.8). Analyses from the CL-bright rims gave age of ca. 580 Ma. No REE analyses were undertaken on this sample due to the absence of garnet.

#### Kozhikodupothai

Three samples (two garnet-leucogranites one with patchy charnockitisation and a garnet-bearing charnockite) were analysed at this location. The garnet leucogranites (IND12-006B and IND12-006C) both gave discordant arrays of analyses with poorly defined upper intercept ages of 2012 ± 53 Ma and 2029 ± 65 Ma and lower intercept ages of 571 ± 31 Ma and 536 ± 48 Ma (Fig. 5e,f). There was some minor zircon rim development in the sample that has the patchy charnockitisation (IND12-006C), analyses of these rims returned a weighted mean age of 524 ± 10 (MSWD = 0.107, n = 3; Fig. 6f). The garnet-bearing charnockite (IND12-006A) yielded an age of 2042 ± 45 Ma with some discordance (Fig. 6f). Two rim analyses from this sample gave ages of 540 ± 24 Ma and 525 ± 14 Ma, within error of the lower intercept age of 536 ± 41 Ma and the lower intercept ages in the garnet-leucogranites (Fig. 6f). REE analysis of zircon and garnet suggests younger zircon rims were in equilibrium with the garnet, whereas the oscillatory-zoned cores show typical igneous patterns and are not in equilibrium with the garnet (Fig. 7c,d).

### Lu–Hf results

**Figure 8:**
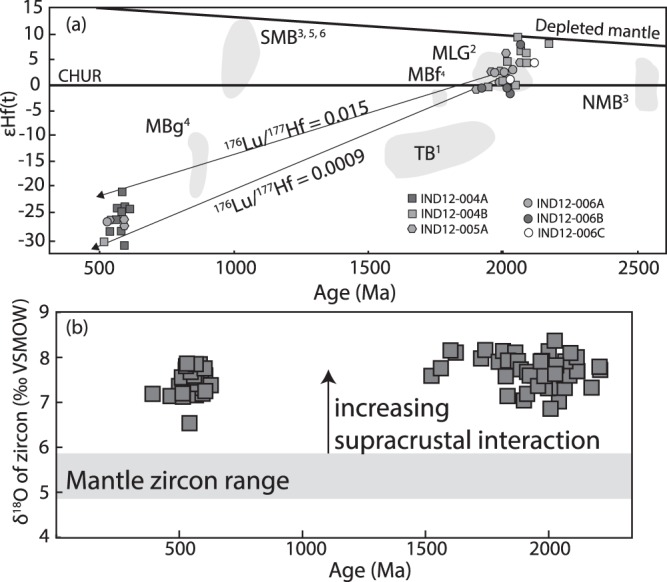
(**a**) Summary of new Lu–Hf data overlain on existing datasets for India, Africa and Sri Lanka (data in Supplementary Table S4). Abbreviations are: TB –Trivandrum Block; MB - Madurai Block with the –f and –g subscripts being felsic gneisses and gabbro respectively; NMB – Northern Madurai Block; MLG – - Mkushi-Luwalizi Gneisses. Data sources include: 1Kroner et al., 2012; 2De Waele et al., 2009; 3Plavsa et al., 2012; 4Teale et al., 2012; 5Plavsa et al., 2014; 6Taylor et al., 2015. (**b**) SIMS O-isotope data from Nagercoil Block zircons (data in Supplementary Table S5).

Hafnium isotopic analyses were carried out on <10% discordant zircon grains and the results are presented in Supplementary Table S4. Data is plotted on epsilon Hf (εHf) vs. age (Ma) plot (Fig. 8a). Two Hf model ages are quoted in Supplementary Table S4, T_DM_ and T_DM_^C^, the latter assumes derivation of magma from average continental crust^28^. The evolution of Lu-Hf in a closed system zircon will be different to that in a piece of crust due to the differing proportions of these elements. On Fig. 8a we plot two evolution lines one for continental crust (Lu/Hf = 0.015) and for the average zircon concentration (Lu/Hf = 0.0009) from a starting point of 2.05 Ga, the age of magmatism in the Nagercoil Block. The younger population of c. 0.55 Ga metamorphic zircon have errors in εHf(T) which overlap both of these evolution lines and therefore the Hf data cannot distinguish between a Pb-loss or the introduction of remelted 2.05 Ga continental crust in these younger grains. The εHf(T) values quoted below are calculated for the corresponding U-Pb ages of each individual analysis.

Hafnium isotopic data from charnockites and leucogranites throughout the Nagercoil Block yield two distinct populations in εHf versus U-Pb age space (Fig. 8a) that reflect cathodoluminescence defined cores and rims. The Palaeoproterozoic cores plot between CHUR and Depleted Mantle (εHf(T) −1.28–9.36), whereas the Ediacaran-Cambrian rims yield εHf(T) values between −31.5 and −20.96.

### Zircon O-isotopes

SIMS oxygen isotope analyses of zircon from all samples falls in the range 7 to 8 per mill (V-SMOW) (Supplementary Table S5; Fig. 8b). There was no difference observed between the concordant Palaeoproterozoic cores and the 580–515 Ma rims (Fig. 8b).

## Discussion

The new U–Pb data constrain the age of the magmatic protoliths of the Nagercoil Block to ca. 2040 Ma (Fig. 6). The Lu–Hf data demonstrate the juvenile nature of this magmatism and support the interpretation that these formed by melting of basaltic source^22^. Oxygen isotope data are consistent with the incorporation of a supracrustal component (Fig. 8b). We interpret the data to indicate the Nagercoil Block represents the remnants of a previously unidentified Palaeoproterozoic magmatic arc. The REE data from zircon, garnet and orthopyroxene show that garnet and orthopyroxene grew in equilibrium with the zircon rims (Fig. 7). This demonstrates that the charnockite assemblage formed during metamorphism at 530 Ma, coinciding with the amalgamation of Gondwana.

Palaeoproterozoic felsic gneisses, which are interpreted to have magmatic protoliths, occur immediately to the northeast within the Trivandrum Block^7,15^. However, the εHf(T) from these rocks are significantly more evolved than the Nagercoil Block data (Fig. 8a). Further north, the Achankovil Zone and southern Madurai Block have recently been interpreted as a Neoproterozoic suture containing Mesoproterozoic to Neoproterozoic juvenile magmatic and metasedimentary rocks^9,21,27^. The northern Madurai Block is composed predominantly of c. 2500 Ma juvenile magmatic rocks and Proterozoic metasediments^21^. Teale *et al*.^29^ reported middle Neoproterozoic gabbro-anorthosites from this region and also minor Palaeoproterozoic felsic gneisses (Fig. 8a).

Sri Lanka, southern Madagascar and eastern Africa lie adjacent to the Nagercoil Block in a reconstructed Gondwana e.g.^12^ (Fig. 1). The Highland Complex of Sri Lanka has been correlated with southernmost India^6,7^. Limited data have been used to suggest magmatic intrusion between 1.90 to 1.85 Ga^30^ and no Hf data are available from central Sri Lanka. In southern Madagascar, 1.79–2.00 Ga magmatic protoliths have been reported by Tucker *et al*.^31^ that have been interpreted to underlie the extensive metasediments in the region^2^. The southeast part of vast Congo Craton, the Bangweulu Block (BB – Fig. 1), lay directly east of southern India/Madagascar in Gondwana (Fig. 1) and is best exposed as the basement to the Irumide Belt of Zambia^32^. These rocks (the Mkushi and Luwalizi gneisses (MLG – Fig. 1)) are deformed 2.04 Ga juvenile orthogneisses that overlap in U–Pb and Hf isotopic composition with the Nagercoil Block samples from this study^32^; Fig. 3. The isotopic similarities between the Irumide basement and the Nagercoil Block rocks provide a strong argument for correlating these regions and assigning the tectonic affinity of southernmost India to Precambrian Africa (Fig. 8a).

Considerable controversy surrounds the tectonic framework of this key orogen in the Gondwana amalgam. Fitzsimons and Hulscher^4^ argued that much of the central EAO originated in Africa and rifted from the Tanzania-Bangweulu continent earlier in the Proterozoic to either collide with India^4^, or back on the African margin at ~650–620 Ma before terminal India-Africa collision at the end of the Ediacaran and into the Cambrian^8^. These models require the existence of multiple oceanic sutures between cratonic India and Africa. In contrast, Tucker *et al*.^2,31^ argue for a Neoproterozoic Greater Dharwar continent and a simple, single suture between ‘Indian crust’ and ‘African crust’ to the west of both India and Madagascar. Boger *et al*.^3^ proposed a modified version of this where southern India and central/eastern Madagascar were also part of Neoproterozoic India, but a microcontinent centred around the Androyen Domain of south-central Madagascar collided first with an arc terrane, preserved in the Vohibory Domain (SW Madagascar), then with Neoproterozoic India.

The data presented here demonstrate that southernmost India has a considerably greater pre-Gondwana affinity with East Africa, than any other block with a magmatic protolith in the central East African Orogen. The major implication of this link is that southernmost India (and Madagascar) is derived from pre-Gondwana Africa and a major strand of the Mozambique Ocean lay to the north-east of the Nagercoil Block. Potential sites of this suture lie in the Palghat-Cauvery shear zone, along the northern margin of the Madurai Block^8^ and within the southern Madurai Block/Achancovil Zone^1^ with the remnants of the Neoproterozoic ocean-basin sediments and associated magmatism preserved^9,21,33^. In addition, the rocks of the Palghat-Cauvery shear zone contain evidence of Neoproterozoic high-pressure metamorphism^34,35^, interpreted ophiolitic rocks^36–38^ and has the geohysical characteristics of a mantle penetrating structure^39^. All of these observations are consistent with the PCSS representing a suture zone along which the remnants of the Mozambique ocean were consumed. In addition, these findings reinforce the notion that presented by various workers and summarized by Collins *et al*.^8^ on the detrital provenance of the Palaeoproterozoic sedimentary units that make up the bulk of the Trivandrum and Madurai Blocks are sourced from African protoliths. The Nagercoil Block could be considered the remnant African basement upon which these sediments were deposited.

The Nagercoil Block was part of the Congo-Tanzania-Bangweulu continent (Africa) that was subsequently welded to India during Gondwana amalgamation where it was metamorphosed to granulite-facies resulting in the formation of orthopyroxene (+/− garnet)-bearing gneisses. The African affinity of southernmost India requires a Neoproterozoic oceanic suture to lie within southern India e.g.^1,8^ rather than in Madagascar e.g.^3^ or to the west of Madagascar^2,31^.

## Methods

### SHRIMP methods, data and standards

Zircon was separated from crushed rock samples using traditional magnetic and methylene iodide heavy liquid separation techniques. Grains were hand picked and mounted in 25 mm diameter epoxy resin discs. Mounts were carbon coated for imaging on a Tescan MIRA3 scanning electron microscope (SEM) with zircon CL images taken at a working distance of 15 mm and using an accelerating voltage of 10 kV. For SHRIMP analyses the samples were coated with a thin membrane of gold that produced a resistivity of 10–15 Ω across the mount surface.

U-Pb isotopes were analysed on the SHRIMP II at the John de Laeter Centre SHRIMP Facility, Curtin University, Perth, Western Australia. The analytical procedures for the Curtin consortium SHRIMP II have been described by^40^ and^41^ and are similar to those described by^42^ and^43^. For zircon analysis a 25–30 μm diameter spot was used, with a mass-filtered O_2_^−^ primary beam of ~2 nA. Data for each spot were collected in sets of 6 scans through the mass range of ^196^Zr_2_O^+^, ^204^Pb^+^, Background, ^206^Pb^+^, ^207^Pb^+^, ^208^Pb^+^, ^238^U^+^, ^248^ThO^+^, ^254^UO^+^. The ^206^Pb/^238^U age standards used were BR266, a Sri Lankan gem zircon^44^, and Temora-2 a zircon grain separate^45^. The ^207^Pb/^206^Pb standard used to enable correction for instrument induced mass fractionation was OG1 zircon^46^. The common Pb correction was based on the measured ^204^Pb^42^. The correction formula for Pb/U fractionation is ^206^Pb^+^/^238^U^+^ = *a*(^238^U^16^O^+^/^238^U^+^)^*b*^ ^47^ using the parameter values of^45^.

External spot-to-spot errors on zircon U-Pb calibration sessions were <1% for both sessions, a minimum error of 1% was applied which reflects the long-term performance of the SHRIMP II facility. Uncertainty cited for individual spot analysis in the text and data tables include errors from counting statistics, the common-Pb correction, and the U–Pb calibration error based on reproducibility of U–Pb measurements of the standard, and are at the 2σ level. Uncertainties of weighted mean values for pooled analyses and upper and lower intercepts in the figures are at the 95% confidence level, with MSWD calculated for concordance and equivalence (Fig. 5). Uncertainty ellipses on concordia diagrams are at the 2σ level (Fig. 5).

### LA-ICPMS method, data and standards

Rare earth element (REE) analyses of zircon and garnet were performed at the Curtin University LA–ICP–MS facility using a Resonetics M-50 193 nm excimer laser with an Agilent 7700 mass spectrometer. Zircon was analysed in the grain separate mount used for SHRIMP analysis, while garnet was analysed in thin section. Beam diameter was 23 μm using a repetition rate of 5 Hz which produced a laser power density of ~3 J/cm^−2^. Data was collected using time resolved data acquisition and processed using the Iolite software package^48,49^. Where appropriate REE values were normalized to chondritic values^50^. Total acquisition time per analysis was 80 s including 30 s of background time and 40 s of sample ablation, followed by a 10 s washout period. Calibration was performed against the NIST 610 standard glass using the coefficients of Pearce, *et al*.^51^. NIST 610 was run 8 times per sample with 3 analyses at the beginning and end and 2 analyses in the middle of each run. Stoichiometric major elements were used for calibration of trace elements in each phase. Stoichiometric Si was used as the internal standardization element for both zircon (14.76%) and garnet (18%). Precision based on repeated analysis of standards is approximately 5–10%, with detection limits for REE in this study ranging from 0.1 to 0.5 ppm. Due to the depth of the laser ablation pit relative to those associated with SHRIMP analysis, several analyses had to be rejected as they intersected heterogeneous material and/or inclusions of other phases.

### Lu-Hf methods, data and standards

Hafnium isotope analyses were conducted on previously dated zircons mounted in epoxy resin using a New Wave/Merchantek LUV213 laser-ablation microprobe, attached to a Nu Plasma multi-collector inductively coupled plasma mass spectrometer (LA-MC-ICPMS). The analyses employed a beam diameter of ∼55 μm and a 5 Hz repetition rate which resulted in ablation pits typically 40–60 μm deep. The ablated sample material was transported from the laser cell to the ICP-MS torch by a helium carrier gas. Interference of ^176^Lu on ^176^Hf was corrected by measurement of interference-free ^175^Lu, and using the invariant ^176^Lu/^175^Lu correction factor 1/40.02669 (DeBievre and Taylor, 1993). Interference of ^176^Yb on ^176^Hf was corrected by measuring the interference-free ^172^Yb isotope, and using the ^176^Yb/^172^Yb ratio to obtain the interference-free ^176^Yb/^177^Hf ratio. The appropriate value of ^176^Yb/^172^Yb was determined through spiking of the JMC475 hafnium standard solution with ytterbium, and finding the value of ^176^Yb/^172^Yb (0.58669) required to yield the ^176^Hf/^177^Hf value for the un-spiked solution. The typical 2σ precision of the ^176^Hf/^177^Hf ratios is +0.00002, equivalent to +0.7 ɛHf unit.

Thirty zircons from the Mud Tank carbonatite locality were analysed, together with the samples, as a measure of the accuracy of the results. Most of the data and the mean ^176^Hf/^177^Hf value (0.282522 ± 0.000015; n = 30) are within 2 standard deviations of the recommended value (0.282522 ± 0.000042 (2σ); Griffin *et al*., 2007). Six analyses of the 91500 zircon standard analysed during this study indicated ^176^Hf/^177^Hf = 0.282320 ± 0.000021 (2σ), which is well within the range of values reported by Griffin *et al*. (2006).

Calculation of initial ^176^Hf/^177^Hf is based on the ^176^Lu decay constant of Scherer *et al*. (2001; 1.867 × 10^−11^ y^−1^) and ɛ_Hf_ values employed the present day chondritic measurement of Blichert-Toft and Albarède, (1997; 0.282772). Calculation of model ages (T_DM_) is based on a depleted-mantle source with (^176^Hf/^177^Hf)_i_ = 0.279718 at 4.56 Ga and ^176^Lu/^177^Hf = 0.0384 (Griffin *et al*., 2004). T_DM_ (crustal) ages were calculated assuming that the Hf within each zircon resided within a reservoir with ^176^Lu/^177^Hf ratio of 0.015, corresponding to an average Continental Crust^28^ (Griffin *et al*., 2002 and Griffin *et al*., 2004).

### O-isotope methods, data and standards

Oxygen isotope ratios (^18^O/^16^O) were determined using a Cameca IMS 1280 multi-collector ion microprobe located at the Centre for Microscopy, Characterisation and Analysis (CMCA), University of Western Australia (UWA). Oxygen isotope analyses were performed with a ca. 3 nA Cs^+^ beam with an impact energy of 20 keV focused to a 10–15 µm spot on the sample surface. Instrument parameters included a magnification of 130 × between the sample and field aperture (FA), 400 μm contrast aperture (CA), 4000 μm FA, 110 μm entrance slit, 500 μm exit slits, and a 40 eV band pass for the energy slit with a 5 eV gap toward the high energy side. Secondary O^−^ ions were accelerated to 10 keV and analyzed with a mass resolving power of approximately 2400 using dual Faraday Cup detectors. A normal-incidence electron gun was used to provide charge compensation and NMR regulation was used for magnetic field control.

Ten seconds of pre-sputtering was followed by automatic centering of the secondary beam in the FA and CA. Each analysis consisted of 20 four-second cycles, which gave an average internal precision of 0.2‰ (2 SE). Analytical sessions were monitored in terms of drift and precision using at least four bracketing standards (Temora II; 8.2‰^52^ every 5–10 sample analyses. Instrumental mass fractionation (IMF) was corrected using Temora II following the procedure described in Kita, *et al*.^53^). The spot-to-spot reproducibility (external precision) was better than 0.3‰ (2 SD) on Temora II during the analytical session. Propagated uncertainty on each *δ*^18^O spot has been calculated by propagating the errors on instrumental mass fractionation determination, including the error on the reference value of the standard, and internal error on each sample data point. The resulting uncertainty was typically between 0.2 and 0.3‰ (2 SD).

## Supplementary information

Supplementary Tables

## References

[1] Santosh M, Maruyama S, Sato K (2009). Anatomy of a Cambrian suture in Gondwana: Pacific-type orogeny in southern India?. Gondwana Research.

[2] Tucker RD, Roig JY, Moine B, Delor C, Peters SG (2014). A geological synthesis of the Precambrian shield in Madagascar. Journal of African Earth Sciences.

[3] Boger SD (2014). From passive margin to volcano–sedimentary forearc: The Tonian to Cryogenian evolution of the Anosyen Domain of southeastern Madagascar. Precambrian Research.

[4] Fitzsimons ICW, Hulscher B (2005). Out of Africa: detrital zircon provenance of central Madagascar and Neoproterozoic terrane transfer across the Mozambique Ocean. Terra Nova.

[5] Collins AS, Santosh M, Braun I, Clark C (2007). Age and sedimentary provenance of the Southern Granulites, South India: U-Th-PbSHRIMP secondary ion mass spectrometry. Precambrian Research.

[6] Braun, I. & Kriegsman, L. M. In *Proterozoic East Gondwana: Supercontinent Assembly and Breakup* Vol. 206 (eds M Yoshida, B Windley, & S Dasgupta) 169–202 (Special Publication of the Geological Society, London, 2003).

[7] Kröner A, Santosh M, Wong J (2012). Zircon ages and Hf isotopic systematics reveal vestiges of Mesoproterozoic to Archaean crust within the late Neoproterozoic–Cambrian high-grade terrain of southernmost India. Gondwana Research.

[8] Collins AS, Clark C, Plavsa D (2014). Peninsular India in Gondwana: The tectonothermal evolution of the Southern Granulite Terrain and its Gondwanan counterparts. Gondwana Research.

[9] Plavsa D (2014). Detrital zircons in basement metasedimentary protoliths unveil the origins of southern India. GSA Bulletin.

[10] Clark C (2015). Hot orogens and supercontinent amalgamation: A Gondwanan example from southern India. Gondwana Research.

[11] Clark C, Fitzsimons ICW, Healy D, Harley SL (2011). How does the continental crust get really hot?. Elements.

[12] Collins AS, Pisarevsky SA (2005). Amalgamating eastern Gondwana: The evolution of the Circum-Indian Orogens. Earth-Science Reviews.

[13] Meert J (2003). A synopsis of events related to the assembly of eastern Gondwana. Tectonophysics.

[14] Schmitt, R. d. S., Fragoso, R. d. A. & Collins, A. S. In *Geology of* Southwest *Gondwana* (eds Siegfried Siegesmund, Miguel A. S. Basei, Pedro Oyhantçabal, & Sebastian Oriolo) 411–432 (Springer International Publishing, 2018).

[15] Kröner A (2015). Palaeoproterozoic ancestry of Pan-African high-grade granitoids in southernmost India: Implications for Gondwana reconstructions. Gondwana Research.

[16] Santosh M, Tagawa M, Taguchi S, Yoshikura S (2003). The Nagercoil Granulite Block, southern India: petrology, fluid inclusions and exhumation history. Journal of Asian Earth Sciences.

[17] Ghosh Joy Gopal, de Wit Maarten J., Zartman R. E. (2004). Age and tectonic evolution of Neoproterozoic ductile shear zones in the Southern Granulite Terrain of India, with implications for Gondwana studies. Tectonics.

[18] Kumar TV (2017). Zircon U-Pb ages and Hf isotopic systematics of charnockite gneisses from the Ediacaran–Cambrian high-grade metamorphic terranes, southern India: Constraints on crust formation, recycling, and Gondwana correlations. GSA Bulletin.

[19] Cenki B, Braun I, Bröcker M (2004). Evolution of the continental crust in the Kerala Khondalite Belt, southernmost India: evidence from Nd isotope mapping, U–Pb and Rb–Sr geochronology. Precambrian Research.

[20] Tomson JK, Rao YJB, Kumar TV, Rao JM (2006). Charnockite genesis across the Archaean-Proterozoic terrane boundary in the South Indian Granulite Terrain: Constraints from major-trace element geochemistry and Sr-Nd isotopic systematics. Gondwana Research.

[21] Plavsa D (2012). Delineating crustal domains in Peninsular India: Age and chemistry of orthopyroxene-bearing felsic gneisses in the Madurai Block. Precambrian Research.

[22] Rajesh HM, Santosh M, Yoshikura S (2011). The Nagercoil charnockite: A magnesian, calcic to calc-alkalic granitoid dehydrated during a granulite-facies metamorphic event. Journal of Petrology.

[23] Taylor RJM (2015). Experimental determination of REE partition coefficients between zircon, garnet and melt: a key to understanding high-T crustal processes. Journal of Metamorphic Geology.

[24] Taylor RJM, Kirkland CL, Clark C (2016). Accessories after the facts: Constraining the timing, duration and conditions of high-temperature metamorphic processes. Lithos.

[25] Johnson TE, Clark C, Taylor RJM, Santosh M, Collins AS (2015). Prograde and retrograde growth of monazite in migmatites: An example from the Nagercoil Block, southern India. Geoscience Frontiers.

[26] Clark C, Collins AS, Santosh M, Taylor R, Wade BP (2009). The *P–T–t* architecture of a Gondwanan suture: REE, U-Pb and Ti-in-zircon thermometric constraints from the Palghat Cauvery shear system, South India. Precambrian Research.

[27] Taylor RJM, Clark C, Johnson TE, Santosh M, Collins AS (2015). Unravelling the complexities in high-grade rocks using multiple techniques: the Achankovil Zone of southern India. Contributions to Mineralogy and Petrology.

[28] Griffin WL (2002). Zircon chemistry and magma mixing, SE China: *In-situ* analysis of Hf isotopes, Pingtan and Tonglu igneous complexes. Lithos.

[29] Teale W (2012). Cryogenian (~830 Ma) mafic magmatism and metamorphism in the northern Madurai Block, southern India: A magmatic link between Sri Lanka and Madagascar?. Journal of Asian Earth Sciences.

[30] Hölzl S, Hofmann AW, Todt W, Köher H (1994). U-Pb geochronology of the Sri Lankan basement. Precambrian Research.

[31] Tucker R (2011). A new geological framework for south-central Madagascar, and its relevance to the “out-of-Africa” hypothesis. Precambrian Res.

[32] De Waele B, Fitzsimons ICW, Wingate MTD, Tembo F, Mapani BSE (2009). The geochronological frameworkof the Irumide Belt of Zambia: A prolongued crustal history along the margin of the Bangweulu Craton. American Journal of Science.

[33] Tomson JK, Bhaskar Rao YJ, Vijaya Kumar T, Choudhary AK (2013). Geochemistry and neodymium model ages of Precambrian charnockites, Southern Granulite Terrain, India: Constraints on terrain assembly. Precambrian Research.

[34] Shimpo M, Tsunogae T, Santosh M (2006). First report of garnet-corundum rocks from southern India: Implications for prograde high-pressure (eclogite-facies?) metamorphism. Earth and Planetary Science Letters.

[35] Collins AS (2007). Passage through India: the Mozambique Ocean suture, high-pressure granulites and the Palghat-Cauvery shear zone system. Terra Nova.

[36] Yellappa T, Chetty TRK, Tsunogae T, Santosh M (2010). The Manamedu Complex: Geochemical constraints on Neoproterozoic suprasubduction zone ophiolite formation within the Gondwana suture in southern India. Journal of Geodynamics.

[37] Sato K, Santosh M, Tsunogae T, Chetty TRK, Hirata T (2011). Subduction-accretion-collision history along the Gondwana suture in southern India: A laser ablation ICP-MS study of zircon chronology. Journal of Asian Earth Sciences.

[38] Santosh M, Xiao WJ, Tsunogae T, Chetty TRK, Yellappa T (2012). The Neoproterozoic subduction complex in southern India: SIMS zircon U-Pb ages and implications for Gondwana assembly. Precambrian Research.

[39] Reddy, P. R. *et al*. In *Tectonics of Southern Granulite Terrain* (ed. M. Ramakrishnan) 79–106 (Geological Society of India Memoir 50, 2003).

[40] De Laeter JR, Kennedy AK (1998). A double focusing mass spectrometer for geochronology. International Journal of Mass Spectrometry.

[41] Kennedy A, De Laeter JR (1994). The performance of the WA SHRIMP II ion microprobe. Eighth Internation Conference on Geochronology, Cosmochronology and Isotope Geology.

[42] Compston W, Williams IS, Meyer C (1984). U-Pb geochronology of zircons from lunar breccia 73217 using a sensitive high mass-resolution ion microprobe. Journal of Geophysical Research.

[43] Williams, I. S. U-Th-Pb geochronology by ion microprobe. *Reviews in Economic Geology*, 1–35 (1998).

[44] Stern RA, Amelin Y (2003). Assessment of errors in SIMS zircon U-Pb geochronology using a natural zircon standard and NIST SRM 610 glass. Chemical Geology.

[45] Black LP (2003). TEMORA 1: A new zircon standard for Phanerozoic U-Pb geochronology. Chemical Geology.

[46] Stern RA, Bodorkos S, Kamo SL, Hickman AH, Corfu F (2009). Measurement of SIMS instrumental mass fractionation of Pb isotopes during zircon dating. Geostandards and Geoanalytical Research.

[47] Claoue-Long, J. C., Compston, W., Roberts, J. & Fanning, C. M. Two Carboniferous ages: a comparison of SHRIMP zircon dating with conventional zircon ages and 40Ar/39Ar analysis. *Geochronology, time scales and global stratigraphic correlation* **54**, 3–21 (1995).

[48] Paton C (2010). Improved laser ablation U-Pb zircon geochronology through robust downhole fractionation correction. Geochemistry, Geophysics, Geosystems.

[49] Paton C, Hellstrom J, Paul B, Woodhead J, Hergt J (2011). Iolite: Freeware for the visualisation and processing of mass spectrometric data. Journal of Analytical Atomic Spectrometry.

[50] Anders E, Grevesse N (1989). Abundances of the elements: Meteoritic and solar. Geochimica et Cosmochimica Acta.

[51] Pearce NJG (1997). A compilation of new and published major and trace element data for NIST SRM 610 and NIST SRM 612 glass reference materials. Geostandards Newsletter.

[52] Black LP (2004). Improved 206Pb/238U microprobe geochronology by the monitoring of a trace-element-related matrix effect; SHRIMP, ID–TIMS, ELA–ICP–MS and oxygen isotope documentation for a series of zircon standards. Chemical Geology.

[53] Kita NT, Ushikubo T, Fu B, Valley JW (2009). High precision SIMS oxygen isotope analysis and the effect of sample topography. Chemical Geology.

